# Papillomavirus-Induced Oncogenesis: Bridging Molecular Mechanisms, Diagnostics, and Global Prevention Strategies

**DOI:** 10.3390/v17040515

**Published:** 2025-04-01

**Authors:** G. Hossein Ashrafi, Mustafa Ozdogan, Muharrem Okan Cakir

**Affiliations:** 1 School of Life Sciences, Pharmacy and Chemistry, Kingston University London, London KT1 2EE, UK; m.cakir@kingston.ac.uk; 2 Division of Medical Oncology, Memorial Hospital, Antalya 07050, Turkey

Human papillomavirus (HPV) has long been recognized for causing benign lesions such as warts, but its pivotal role in cancer was established in the late 20th century, culminating in the 2008 Nobel Prize awarded to Harald zur Hausen for linking HPV to cervical cancer [1]. Today, persistent HPV infection is known to cause nearly 5% of all cancers worldwide [2]. Cervical cancer, in particular, is virtually HPV-dependent—it is the fourth most common cancer in women globally, with 604,000 new cases and 342,000 deaths in 2020, and about 70% of cases are caused by HPV16 and HPV18 [3,4]. Oncogenic HPVs (over a dozen “high-risk” types) also contribute to a significant fraction of other malignancies, including anogenital cancers (anal, vulvar, vaginal, penile) and head and neck cancers (especially oropharyngeal) [5]. The recognition of HPV as a major oncogenic virus has driven profound advances in prevention—notably, prophylactic vaccines and HPV-based screening—and spurred extensive research into the molecular biology of HPV-induced carcinogenesis. Despite these advances, HPV-related cancers remain a pressing global health challenge, especially in low-resource settings, underscoring the need for continued research and public health action.

In light of the importance of HPV in oncology, this Special Issue of *Viruses* (“Papillomavirus-Induced Oncogenesis: Current Insights and Future Directions”) was dedicated to exploring the latest insights into how HPVs cause cancer and how to combat these cancers. Below, we outline the scope of this Special Issue and summarize the contributions of each article, which collectively span molecular mechanisms, diagnostic innovations, epidemiological studies, therapeutic strategies, and prevention efforts. Together, these articles enrich our understanding of HPV-induced oncogenesis across multiple anatomical sites and inform future directions in research, clinical practice, and public health.

This Special Issue aims to provide a comprehensive platform for examining papillomavirus-induced oncogenesis from bench to bedside. Contributions were invited across a broad range of topics, including but not limited to the following:

- Molecular and cellular mechanisms: elucidation of how HPVs drive malignant transformation and immune evasion in diverse anatomical sites;
- Diagnostics and biomarkers: advances in tools and techniques for detecting HPV infections and HPV-associated premalignant or malignant lesions;
- Epidemiology and burden: studies analyzing the incidence, risk factors, and trends of HPV-associated cancers, yielding insights into their global public health impact;
- Therapeutics and clinical management: development of innovative treatments (e.g., immunotherapies, targeted therapies) and identification of biomarkers for personalized management of HPV-related cancers;
- Prevention strategies: evaluation of preventive measures, particularly HPV vaccination programs and screening interventions, and their effectiveness in reducing cancer risk across different populations.

By bringing together research and reviews on these themes, this Special Issue intends to foster a holistic understanding of HPV oncogenesis and to highlight opportunities for intervention—from molecular targets for therapy to implementation of vaccinations and screening in populations at risk.

The Special Issue features a collection of eleven peer-reviewed papers (research articles, short communications, and reviews) that fulfill these aims. Key findings from each contribution are summarized below:

1. HPV16 E6 and immune evasion (Konstantopoulos et al., 2024): This study dissects a molecular pathway via which the HPV16 E6 oncoprotein enables cervical cancer cells to evade immune surveillance [6]. Using cervical cell lines with integrated HPV16, the authors knocked out the E6 gene via CRISPR/Cas9 and observed striking changes in the regulation of the immune checkpoint machinery. The loss of E6 led to the upregulation of microRNA-143 and the concomitant downregulation of its target HIF-1α, a transcription factor that drives PD-L1 expression. Accordingly, E6 knockout cells exhibited reduced PD-L1 on the cell surface. These findings implicate the HPV16 E6–miR-143–HIF-1α axis as a key regulator of PD-L1 levels in cervical carcinoma cells, shedding light on how HPV oncoproteins foster an immunosuppressive tumor microenvironment. Identifying this E6-mediated immune evasion mechanism is important for research into immunotherapies; it suggests that targeting the E6 oncoprotein or its downstream pathway could enhance immune recognition of HPV-transformed cells [6].
2. Adopting molecular screening in Mexico (Castrillo-Díez et al., 2024): In this original article from a private gynecological practice in Mexico, researchers evaluated the utility of replacing or augmenting traditional Pap smear screening with molecular diagnostic tools [7]. Over 4499 cervical samples were split for parallel testing: Pap cytology, high-risk HPV DNA PCR, and p16/Ki-67 dual immunocytochemistry as a biomarker of transforming infections. The study revealed significant discordances between cytology and HPV testing. Notably, 25.1% of cases interpreted as low-grade cytological lesions and 47.9% of high-grade cytology cases were HPV-negative via PCR, suggesting possible overcalls by cytology. Conversely, 22.1% of Pap-negative cases were HPV-positive, indicating missed infections. Importantly, among HPV-positive women, one-third showed p16/Ki-67 dual-stain positivity, flagging those with likely clinically relevant infections. The authors conclude that incorporating HPV DNA testing and triage with biomarkers (such as p16/Ki-67) can improve accuracy—for example, dual staining after a positive HPV test could prevent unnecessary colposcopies in women without true precancer. However, they also encountered challenges, chiefly a deep-rooted reluctance among patients and some clinicians to move away from the conventional Pap/colposcopy paradigm. This study’s findings support the adoption of HPV-based screening in middle-income settings and highlight the need for education and policy change to overcome sociocultural barriers in cervical cancer prevention [7].
3. HPV genital warts and risk factors in Nigeria (Ogbolu et al., 2024): This epidemiological study investigated self-reported HPV infection and genital warts among university students and staff in northern Nigeria, offering insight into awareness and risk factors in a setting without free HPV vaccination. Using an online survey (*n* ≈ 400 participants), the authors assessed respondents’ sexual behavior, HPV knowledge, and history of HPV infection or warts. The majority were young adults, and 81.3% of those reporting HPV16/18 infection were aged 26–40, suggesting higher exposure or diagnosis in this age group [8]. Interestingly, being single or divorced (not in a sexually active relationship) was also associated with having reported an HPV infection, which may reflect higher risk behaviors or healthcare-seeking patterns in these groups. Logistic regression analysis for predictors of genital warts indicated that individuals aged 26–40 had lower odds of reporting warts than younger participants (OR ~0.45), consistent with the idea that the peak incidence of visible warts occurs in younger adults. Overall, the study found gaps in HPV knowledge and low vaccine uptake, unsurprising given the lack of a free immunization program. The authors urge implementation of HPV awareness campaigns and the introduction of accessible vaccination at the university and community level. This study underscores the public health importance of education and vaccination in low- and middle-income countries, where the HPV-related disease burden remains high [8].
4. Barriers to cervical cancer prevention in Saudi Arabia (Moshi et al., 2024): This short communication provides a public health overview of the challenges in scaling up HPV vaccination and cervical screening in the Kingdom of Saudi Arabia (KSA). Despite the availability of effective HPV vaccines in KSA, the uptake remains strikingly low—only about 7.6% of eligible women have received the vaccine [9]. The authors compiled evidence from the literature and national reports to map the reasons for this low coverage. They found that while awareness of cervical cancer risks exists, there are persistent misconceptions and low public knowledge about the HPV vaccine’s benefits. Cultural factors and the lack of routine screening programs contribute to a situation where cervical cancer, although less common than in some other regions, could potentially rise if preventive measures are not widely adopted. The article calls for comprehensive community-level knowledge, attitude, and practice (KAP) interventions to dispel myths and increase acceptance of HPV vaccination. It also highlights the need to strengthen cancer registry systems in KSA to better track cervical cancer incidence and outcomes. By identifying these barriers and proposing solutions—such as education campaigns and improved surveillance—this communication provides actionable insights for public health authorities. Its implications extend beyond KSA, as many regions with traditionally low cervical cancer incidence may face similar hurdles in the era of HPV vaccination [9].
5. HPV in lung cancer—a regional study from Turkey (Alikanoğlu and Karaçay, 2024): In an effort to broaden the understanding of HPV’s oncogenic scope, this study examined the presence of high-risk HPV in lung cancers from patients in the Mediterranean region of Turkey [10]. Fifty lung tumor specimens (primarily non-small cell lung cancers) and seven non-cancerous lung tissue controls were analyzed via PCR for HPV DNA (with genotyping) and via immunohistochemistry for the surrogate marker p16 and for EGFR protein expression. Remarkably, HPV DNA was detected in 39 of 50 (78%) lung cancers. HPV51 was the most frequent type identified, followed by the canonical high-risk type HPV16. p16, which is often overexpressed in HPV-driven tumors, was positive in 24% of the lung cancers; all p16-positive cases were also HPV-positive. However, the majority of HPV-positive tumors (27 cases) were p16-negative, and overall, no statistically significant correlation was found between HPV status and p16 expression, patient age, sex, or smoking history. The control (non-neoplastic) lung tissues had a lower frequency of HPV (not significant given the small number). These results add to the ongoing debate about HPV’s role in lung cancer. The high proportion of HPV DNA-positive cases in this Turkish cohort suggests a possible etiological or co-factor role, at least regionally. However, the lack of p16 concordance and the epidemiologic confounders (e.g., smoking in many patients) underscore that HPV in lung cancer may not follow the same paradigm as in cervical or head-neck cancers. The authors call for further studies to clarify HPV’s role in lung carcinogenesis. For clinicians, this work raises awareness that HPV might be present in unexpected sites, potentially affecting how we interpret molecular results or consider preventive strategies (e.g., could HPV vaccination have any impact on lung cancer in never-smokers?). More broadly, it highlights the need for global research on virus-associated cancers beyond the traditionally recognized sites [10].
6. HPV and male urogenital cancers—a comprehensive review (Karaoğlan and Ürün, 2024): While cervical cancer prevention has been a central focus of HPV research, this review shifts attention to HPV’s role in urogenital cancers in men [11]. The authors provide a thorough overview of HPV-related carcinogenesis in male anatomic sites, including penile cancer, which is clearly linked to high-risk HPV, as well as emerging evidence for HPV associations with prostate and bladder cancers. A recurring theme is the complexity of studying HPV in men: despite the substantial prevalence of HPV infections among men, there are no approved screening tests for male HPV infection, and routine testing in asymptomatic men is not performed. The review compiles epidemiological data showing geographic variation in the fraction of male genitourinary cancers attributable to HPV (for instance, HPV is found in a large subset of penile squamous cell carcinomas, but the relationship in prostate cancer remains contentious). The authors also discuss the molecular mechanisms by which HPV might contribute to urogenital oncogenesis—such as E6/E7 oncoprotein effects in penile lesions—and note the differences in viral carcinogenesis between sexes and organ sites. A key insight is that while HPV-driven cervical carcinogenesis is well understood, more research is needed to elucidate HPV’s role in male malignancies. The review underscores the importance of vaccinating boys (not just girls) to confer direct protection against HPV-related cancers in men, which is especially relevant for countries that have recently expanded vaccination programs to both sexes. In summary, Karaoğlan and Ürün highlight both the knowns and unknowns of HPV in male cancer pathology, calling for heightened attention to male-focused HPV research and preventive strategies to reduce the burden of urogenital cancers worldwide [10].
7. Global progress and challenges in cervical cancer prevention (Castle, 2024): Philip E. Castle’s review provides a broad perspective on worldwide cervical cancer control, reflecting on past successes and future hurdles [12]. The review opens by acknowledging that, despite the Pap smear revolution in the mid-20th century, cervical cancer persists as a leading cause of cancer death in women, especially in low-income regions. The identification of HPV as the necessary cause of cervical cancer transformed prevention strategies, giving us two powerful tools: prophylactic HPV vaccination and HPV DNA testing for screening. Castle discusses how these innovations led to the World Health Organization’s call for the elimination of cervical cancer as a public health problem, with ambitious targets for 2030 (90% of girls vaccinated by age 15, 70% of women screened with a high-performance test by age 35 and again by 45, and 90% of cervical disease adequately treated). The review celebrates the progress to date—for instance, dramatic declines in cervical cancer in some countries that have achieved high vaccine coverage—but it focuses on the “moving forward” aspect: the critical challenge of ensuring equity. A looming concern is that the gap between high-resource and low-resource settings could widen if vaccine roll-out and screening programs are not supported in poorer regions. Castle identifies obstacles such as cost, healthcare infrastructure, and vaccine hesitancy that must be overcome to avoid exacerbating disparities. Additionally, the review addresses next-generation technologies (such as self-sampling for HPV testing, novel biomarkers for triage, and potential therapeutic vaccines) and how they might be leveraged to improve prevention and early detection. This comprehensive overview serves as a roadmap for global health policy on HPV. It emphasizes that while cervical cancer could be largely prevented, success will require a concerted international effort, funding, and political will to implement known interventions broadly and equitably [12].
8. HPV co-infections with other STIs in cervical screening (Latorre-Millán et al., 2025): This prospective cohort study from Spain investigated the prevalence of other sexually transmitted infections (STIs) in women who tested positive for high-risk HPV, to inform integrated screening approaches [13]. The authors followed 254 women ages 25–65, all of whom had an HR-HPV infection detected and no history of cervical cancer and screened them for four common non-HPV STIs: *Chlamydia trachomatis*, *Neisseria gonorrhoeae*, *Mycoplasma genitalium*, and *Trichomonas vaginalis*. Overall, they found that co-infections were relatively infrequent: only 5.1% of HPV-positive women had any of these STIs (compared to 3.8% of HPV-negative women in a control group). However, in the subgroup of younger women (25–34 years) with persistent HPV infection, the prevalence of an STI co-infection was significantly higher (20.0%) than in HPV-positive women without persistence (4.2%). Moreover, multiple concurrent infections (HPV with more than one other pathogen) were detected in some HPV-positive individuals, whereas STI occurrences in HPV-negative women were usually single pathogens when present. These data suggest that women with persistent high-risk HPV, especially at younger ages, may be at elevated risk for other STIs—possibly due to shared risk factors or biological susceptibility. The study’s implication is that screening programs might consider offering a broader panel of STI tests when a woman is found to be HPV-positive, particularly in high-risk groups, as a way to improve women’s sexual health outcomes holistically. Additionally, treating co-infections such as chlamydia might indirectly benefit HPV management, since coinfections and inflammation could influence HPV persistence or progression. Latorre-Millán et al. contribute to the epidemiological understanding that cervical cancer prevention does not happen in isolation: integrating STI prevention and treatment can be an important component of comprehensive cervical cancer control.
9. HPV and upper gastrointestinal cancers—a review (Özozan et al., 2025): Expanding the discussion of HPV beyond the typical anogenital and oropharyngeal sites, this review examines evidence for HPV’s involvement in upper gastrointestinal (UGI) tract cancers, with a focus on esophageal cancer [14]. Esophageal squamous cell carcinoma (ESCC) has been studied for possible links to HPV, given some histopathological similarities to head and neck squamous cancers. Özozan et al. summarize a body of research indicating that HPV DNA can be found in a subset of esophageal cancers, though prevalence estimates vary widely by region (with some high-incidence areas of ESCC showing HPV in a notable fraction of tumors, while other studies find little to no HPV). The review discusses the oncogenic mechanisms through which HPV might contribute to UGI carcinogenesis, such as via the action of E6/E7 in esophageal epithelial cells, and reviews studies that have attempted to correlate HPV presence with clinical outcomes. Notably, the authors point out that if a causative role for HPV in a subset of esophageal cancers is confirmed, it could have implications for prevention and therapy. For example, HPV vaccination might eventually contribute to reducing not only cervical cancer but also any HPV-associated esophageal cancers, and HPV-targeted treatments (such as the use of immune checkpoint inhibitors or therapeutic vaccines) could benefit patients with HPV-positive esophageal tumors. However, the review is cautious to note that, unlike cervical cancer, UGI cancers have multifactorial etiologies (tobacco, alcohol, dietary factors, etc.), and HPV’s role is still under investigation. By compiling current data, this article helps clarify that while HPV is a well-established agent in certain cancers, its presence in other less common sites such as the upper GI tract requires further study. It encourages oncologists and researchers to remain open-minded about HPV’s oncogenic reach and to continue investigating whether a preventive HPV vaccine might have broader cancer prevention benefits than currently realized [14].
10. Single-cell transcriptomic atlas of HPV-positive vs. HPV-negative head and neck cancers (Bedard et al., 2025): In this cutting-edge research article, investigators applied single-cell RNA sequencing (scRNA-seq) to compare the cellular landscapes of HPV-positive and HPV-negative head and neck squamous cell carcinomas (HNSCCs) [15]. It is well known that HPV-positive HNSCC (mostly oropharyngeal tumors) behave differently from HPV-negative HNSCC (often linked to smoking/alcohol)—for instance, HPV-positive cases generally respond better to therapy and have a distinct risk profile. Bedard et al. sought to characterize the differences in tumor cell subpopulations between these two cancer subsets, hypothesizing that distinct epithelial tumor cell programs might underlie their divergent clinical behaviors. By analyzing public scRNA-seq datasets comprising thousands of single cells, they identified and annotated epithelial subpopulations within the tumors based on gene expression profiles. The study revealed transcriptomic signatures and ontologies for several epithelial cell clusters, some of which were enriched in HPV-positive cancers and others in HPV-negative cancers. For example, certain differentiation states or stress-response phenotypes of tumor cells were more prominent in one subtype versus the other. They also pinpointed candidate biomarkers—genes uniquely upregulated in HPV-associated tumor cells or vice versa—that could serve as potential therapeutic targets or prognostic markers. The implications of this work are significant: by providing a high-resolution atlas of tumor cell heterogeneity in HNSCC, it lays the groundwork for developing more tailored treatments. One major goal in the field is to de-escalate therapy for HPV-positive HNSCC (to reduce toxicity) without compromising efficacy and, conversely, to find better treatments for HPV-negative HNSCC, which tends to be more aggressive. The distinct epithelial cell populations identified by Bedard et al. offer new insights into the biology of these tumors. In the future, clinicians might use markers of these subpopulations to stratify patients or to guide targeted therapies that address the specific tumor ecology of HPV-positive vs. HPV-negative cancers. This study exemplifies how advanced genomics can deepen our understanding of viral oncogenesis and ultimately improve clinical management [15].

Collectively, the articles in this Special Issue underscore both the breadth and depth of current research on HPV-induced cancers. From basic molecular oncology to public health science, these studies illustrate a unifying theme: tackling HPV-associated malignancies requires an integration of insights across the continuum from bench to bedside. In terms of remaining gaps, several directions for future research and action emerge from these articles. First, there is a need for continued investigation of the molecular crosstalk between HPV and host cells. For example, the discovery of the miR-143/HIF-1α pathway in HPV-mediated immune evasion invites further exploration into therapeutic interventions that could restore immune detection of HPV-positive tumor cells. Second, implementation science will play a critical role in translating advances into impact: studies such as Castrillo-Díez et al. and Moshi et al. illustrate that having effective technologies (HPV tests, vaccines) is not enough—we must also address healthcare delivery, education, and policy to ensure these tools reach the people who need them. This may involve tailoring programs to local contexts, training healthcare providers, subsidizing costs, and vigorously combatting misinformation about HPV and vaccines. Third, there is room for innovation in therapeutics. As HPV-associated cancers become more common in certain domains (e.g., oropharyngeal cancers are rising in incidence in many countries), developing treatments that specifically exploit the virus’s presence is an appealing strategy. Immunotherapies that target HPV antigens (such as E6/E7 therapeutic vaccines or T-cell therapies) and de-escalation trials for HPV-positive cancers are ongoing and should be informed by the kind of biological data presented in this Special Issue (such as the unique tumor cell subsets in HPV-positive HNSCC). Finally, continued surveillance and research are needed to monitor the long-term effects of vaccination programs—not only on cervical cancer rates but also on other HPV-related diseases. As more cohorts of vaccinated individuals age, we expect changes in the epidemiology of HPV infections and potentially a reduction in some cancers; robust data will be crucial to guide future vaccine policy (e.g., number of doses, coverage of non-16/18 types, and the inclusion of boys).

In conclusion, the contributions in this Special Issue highlight remarkable progress in our understanding of HPV-related cancers and bring to light the multifaceted approach required to combat them. HPV-driven cancers exemplify a success story in biomedical science—from discovering a viral cause, to developing preventive vaccines, to now tailoring precision approaches to diagnosis and treatment—but they also exemplify the challenges of ensuring that scientific advances translate into public health benefits for all. By integrating molecular research with epidemiology and prevention, as showcased in this Special Issue, we move closer to a future in which the cancers caused by HPV are significantly reduced, if not eliminated. The insights and strategies discussed here are valuable stepping stones toward that global goal. Continued interdisciplinary collaboration between virologists, oncologists, public health experts, and policymakers will be essential to fully capitalize on these advances and to fill the remaining gaps in knowledge and practice. The fight against HPV-induced cancers is far from over, but with the concerted efforts reflected in these studies, there is ample reason for optimism that we can outpace this virus through science and public health action.
